# Liquid biopsy-based early tumor and minimal residual disease detection**: New perspectives for cancer predisposition syndromes**

**DOI:** 10.1515/medgen-2023-2049

**Published:** 2023-12-05

**Authors:** Lena Bohaumilitzky, Johannes Gebert, Magnus von Knebel Doeberitz, Matthias Kloor, Aysel Ahadova

**Affiliations:** Institute of Pathology University Hospital Heidelberg Heidelberg Germany; University Hospital Heidelberg Department of Applied Tumor Biology, Institute of Pathology Heidelberg Germany; Heidelberg University Hospital Department of Applied Tumor Biology, Institute of Pathology Heidelberg Germany; Institute of Pathology University Hospital Heidelberg Heidelberg Germany; Heidelberg University Hospital Department of Applied Tumor Biology, Institute of Pathology Heidelberg Germany

**Keywords:** Liquid biopsy, cancer predisposition syndromes, clinical management, early cancer detection, Lynch syndrome

## Abstract

Genetic predisposition is one of the major measurable cancer risk factors. Affected patients have an enhanced risk for cancer and require life-long surveillance. However, current screening measures are mostly invasive and only available for certain tumor types. Particularly in hereditary cancer syndromes, liquid biopsy, in addition to monitoring therapy response and assessing minimal residual disease, holds great potential for surveillance at the precancerous stage and potentially even diagnostics. Exploring these options and future clinical translation could help reduce cancer risk and mortality in high-risk individuals and enhance patients’ adherence to tailored surveillance protocols.

## Glossary

CPS Cancer predisposition syndrome
MMR Mismatch repair
MRI Magnetic resonance imaging
LS Lynch syndrome
CRC Colorectal cancer
LB Liquid biopsy
CTC Circulating tumor cell
EV Extracellular vesicle
cfDNA/ctDNA Circulating cell-free/Circulating tumor DNA
cfRNA/ctRNA Circulating cell-free/Circulating tumor RNA
ICB Immune checkpoint blockade
MRD Minimal residual disease

## Cancer predisposition syndromes and their clinical management

Since the first descriptions of genetic predisposition to cancer by Paul Broca, who observed a clustered occurrence of breast cancer in his wife’s family, more than 100 different cancer predisposition syndromes (CPSs) have been described [1]. The majority of cancer predisposition genes present with an autosomal dominant inheritance pattern and act as classical tumor suppressors, requiring a somatic inactivation of the second remaining wild type allele following the Knudson’s two-hit hypothesis [2]. Prevalent cancer predisposition genes have been shown to be critical components of pathways maintaining genomic stability (e. g. *BRCA* and mismatch repair (MMR) genes) and controlling cellular proliferation (e. g. *APC*, *TP53*, *RB1*) [3]*.*

Overall, 5–10 % of all cancers are attributable to CPSs which are generally characterized by an accelerated rate of cancer manifestation in different organ systems, often with an age of onset younger than 50 years, and frequent synchronous and metachronous manifestation [4,5].

Due to the increased risk of developing cancer, effective and regular clinical surveillance of affected individuals and families with CPSs is crucial to facilitate early cancer detection which is pivotal for improving treatment options and survival. Different surveillance protocols for CPSs have been established which mainly encompass routine physical examination, imaging as well as biochemical/metabolic assays. For example, cancer surveillance for hereditary breast cancer comprises palpation examination of the breast, breast ultrasound, mammography and magnetic resonance imaging (MRI) [6] and guidelines for the management of Lynch syndrome recommend regular colonoscopy [7,8]. Risk-reducing surgery is another pillar in the care of individuals with CPSs, particularly for cancers with high lifetime risk and poor prognosis. Depending on guidelines, prophylactic surgical interventions can be recommended, e. g., mastectomy and salpingo-oophorectomy in *BRCA* variant carriers [6,7]. However, structured surveillance strategies also place a burden on patients and are associated with multiple drawbacks. For instance, Li-Fraumeni carriers are recommended to follow the comprehensive ‘Toronto protocol’ which encompasses a combination of routine physical exams, abdominal and pelvic ultrasound, MRI, mammography, colonoscopy and regular blood tests. Importantly, Li-Fraumeni carriers under surveillance present with a significant survival advantage [9], but several risks are associated with such close-meshed cancer surveillance. The use of MRI imaging often requires contrast agents, such as the widely applied gadolinium-based agents, which have been associated with adverse effects and toxicity upon recurrent exposure [10]. Further, MRI screening often requires sedation or general anesthesia in pediatric patients which poses additional risks such as respiratory events [11]. Moreover, radiological imaging, e. g. mammography, is accompanied by radiation risk exposure. Even though the risk of radiation-induced carcinogenesis is often outweighed by the potential benefits of the screening, studies suggest that caution is required with individuals with an inherited cancer predisposition or hyper-radiosusceptibility [12].

**Table 1:**
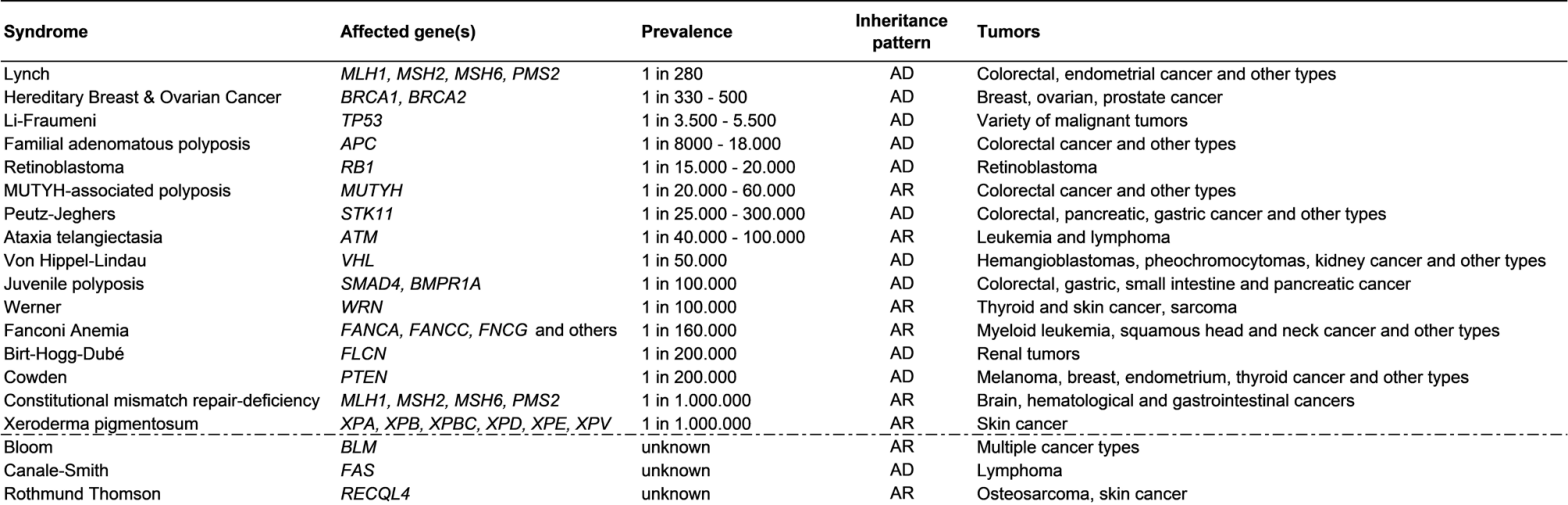
List of cancer predisposition syndromes with their key features sorted by descending prevalence reported in the general population. AD: autosomal dominant; AR: autosomal recessive *[4,5]*.

The described drawbacks for screening approaches as well as the lack of effective surveillance for several CPS and/or associated cancer types warrant further research to improve the clinical management of affected individuals.

## Lynch syndrome – the most common inherited cancer syndrome

The most common CPS with autosomal dominant inheritance is Lynch syndrome (LS) which is characterized by a pathogenic germline variant in one of the MMR genes (*MLH1*, *MSH2*, *MSH6*, *PMS2*). LS carriers are predisposed to the development of MMR-deficient/microsatellite-unstable (MSI) cancers in different organ systems, particularly in the colorectum and endometrium [13]. Clinical surveillance of LS carriers is paramount to reduce cancer risk and facilitate early cancer detection. However, colorectal surveillance by regular colonoscopy is the only protocol proven to be effective. Currently, in most countries, including Germany, colonoscopy at least every 24 months is recommended for LS carriers [7,8]. However, even under regular colonoscopy LS carriers have an up to 15 % 10-year risk of developing colorectal cancer (CRC). Several hypotheses have been proposed to explain the limited effectiveness of colonoscopic surveillance in LS. These include an accelerated progression from adenomas to carcinoma in LS carriers which may render the timeframe for colonoscopic detection of precancerous lesions too short, leading to incident CRC occurrence. In contrast to sporadic adenomas with a dwell time of around 10 years, LS-associated adenomas can undergo rapid malignant transformation, suggested to be fueled by MMR deficiency, within 3–5 years. However, no difference in CRC incidence has been identified between different colonoscopy intervals (annual, 2- and 3-yearly) and the lack of efficacy of shorter intervals points towards an adenoma-free progression of some cancers [14]. Such an alternative route to CRC development may start with LS-specific MMR-deficient premalignant lesions in the colonic mucosa of LS carriers [15]. Importantly, these lesions are mostly indistinguishable from normal colonic crypts and thus not detectable by conventional colonoscopy, offering an explanation for the occurrence of LS-associated CRC even under short-interval colonoscopy surveillance [14].

Apart from CRC, LS presents with a broad tumor spectrum comprising endometrial, ovarian, stomach, kidney, bladder, pancreatic and brain cancer. However, for most of them no surveillance approaches are available or evidence on the value of surveillance is insufficient and no standard surveillance protocols are in place [16]. Importantly, a molecular hallmark and biomarker of LS-associated cancers is the MSI phenotype which opens doors for organ-independent LS surveillance by systemic, liquid biopsy-based MSI detection.

## Liquid biopsy – analytes and applications

Liquid biopsy (LB) incorporates the analysis of liquid samples obtained from different bodily fluids such as blood, urine, saliva, pleural effusions and cerebrospinal fluid [17]. Compared to conventional tissue-based biopsies, which are invasive, sometimes associated with significant procedural risk, time consuming and limited by inaccessibility of the tissue of interest as well as tumor heterogeneity, LB offers several advantages. The non- or minimally-invasive nature of LB enables sampling with minimal risk and, thus, repeated sampling. Further, the broad diversity of LB analytes provides vast information on the underlying tumor. LB analytes include circulating tumor cells (CTCs), circulating nucleic acids, extracellular vesicles (EVs), proteins, lipids and metabolites [17].

CTCs are defined as tumor cells that enter the circulatory or lymphatic system upon being sloughed from primary and/or metastatic tumors. CTCs have been shown to play a crucial role in cancer metastasis as a limited number of CTCs survive sheer stress and the host’s immune system in the periphery to infiltrate and colonize distant organs [18]. Clinically, CTCs represent a valuable tumor surrogate in the blood stream and have the potential to serve as real-time ‘snapshot’ of the tumor. Quantification, molecular phenotyping and genome, transcriptome as well as proteome analyses of CTCs can provide significant insight into disease progression and therapy efficacy. Multiple studies have successfully demonstrated the prognostic value of CTCs [19]. For instance, it could be shown that the mere number of CTCs in patients with different cancer types has prognostic value and higher CTC counts were generally associated with a higher risk of metastasis [20–22] and poorer prognosis [23–26]. Further, a decrease or full clearance of CTCs was found to be indicative of a good therapeutic response [27,28]. Currently, the clinical application of CTCs is still limited by their challenging isolation and characterization. Particularly at an early tumor stage the use of CTCs is severely restricted by their scarcity. So far the CellSearch^TM^ test system is the only method for CTC detection that has been cleared by the US Food and Drug Administration (FDA) [29] and CTCs have not been included in standardized clinical guidelines yet [19].

Circulating nucleic acids as LB analytes comprise circulating cell-free DNA (cfDNA) including the tumor-derived fraction of circulating tumor DNA (ctDNA) and circulating cell-free/tumor RNA (cfRNA/ctRNA). Particularly cfDNA/ctDNA has been extensively studied with regards to clinical applications [30–32]. CtDNA is shed from tumor cells via various mechanisms including apoptosis, necrosis and other types of cell death. Importantly, ctDNA reflects the molecular makeup of the underlying tumor and virtually all cancer-associated alterations in coding and noncoding regions can be detected in ctDNA [33]. CtDNA has shown potential for versatile applications such as cancer diagnosis, prognosis and therapy response monitoring. In contrast to CTCs, which currently have a limited use for cancer detection, a cfDNA/ctDNA-based test for colorectal cancer screening assessing the methylation status of the Septin9 promoter (Epi proColon by Epigenomics AG) has already been approved by the FDA [34]. Further, the prognostic value of ctDNA has been demonstrated in various studies. For instance, the absence of ctDNA after surgery and/or chemotherapy was associated with a better patient outcome and a smaller chance of recurrence [35,36]. Elevated ctDNA concentration and changes in its mutational profile were also found to be indicative of treatment failure in patients with different cancer types [35,37–41]. Moreover, sequencing ctDNA has been successfully applied for the selection of treatment illustrated by the FDA approval of the cobas EGFR Mutation Test v2 (Roche Molecular Diagnostics) for the selection of tyrosine kinase inhibitors in non-small cell lung carcinoma patients [42].

EVs are a heterogeneous group of membrane-enclosed particles secreted into the extracellular space by virtually all cells. EVs play a crucial role in intercellular communication by transporting a plethora of different cargo molecules including nucleic acids, proteins and lipids between donor and recipient cells. Importantly, the EV cargo composition reflects the physiological/pathological state of the donor cell [43]. Numerous studies have demonstrated the diverse roles of EVs and EV-mediated functional communication in different phases of tumorigenesis [44,45]. Tumor-derived EVs reflect the malignant status of the donor cell and carry tumor-specific cargo such as oncoproteins, genomic DNA/oncogenes and oncogenic microRNAs (miRNA). The variety of cancer-associated molecules render EVs a valuable source of cancer biomarkers and can provide complex information on the tumor [45]. While the release of CTCs and ctDNA is associated with metastatic dissemination and cell death, respectively, EVs are actively shed by live cells. Thus EVs might be the earliest biomarkers detectable during cancer development and most suited for diagnostic purposes. Indeed, several EV-associated molecules have shown promise in early detection of different cancer types. For example, melanoma patients displayed significantly higher levels of plasma EV-associated CD63 and caveolin-1, compared to healthy individuals [46]. Further, a high abundance of CD147 and CD9 double positive serum EVs was found to be characteristic for CRC patients [47]. Moreover, multiple studies suggest a suitability of EVs and their versatile cargo for prognostic applications and therapy response monitoring [48–51]. Nevertheless, to this day no EV-associated biomarker has been approved by national or international agencies [52].

**Figure 1:**
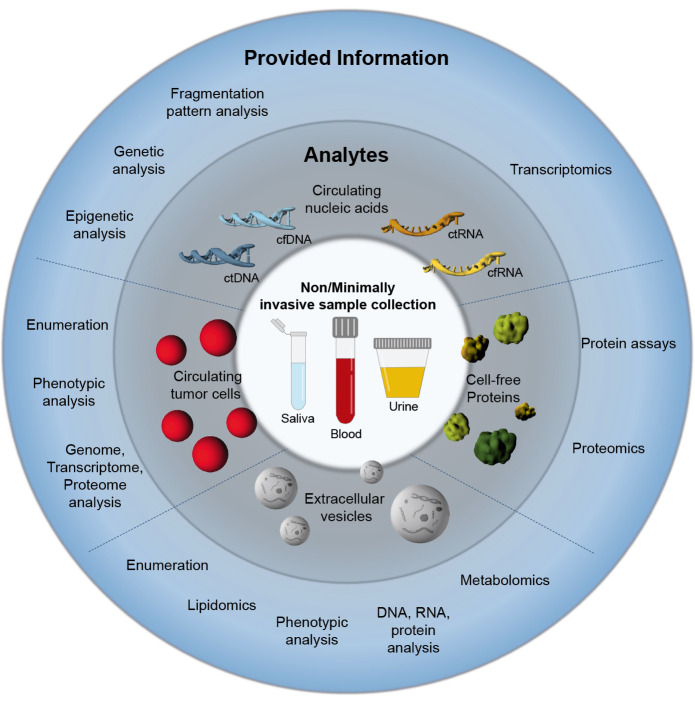
Graphical overview on liquid biopsy, its analytes and obtainable information on the tumor. Parts of the figure were drawn by using pictures from Servier Medical Art. Servier Medical Art by Servier is licensed under a Creative Commons Attribution 3.0 Unported License (https://creativecommons.org/licenses/by/3.0/).

In comparison to LB analytes described above, circulating proteins as cancer biomarkers are more established in the clinical routine. Examples of FDA-approved markers are the prostate-specific antigen (PSA) for prostate cancer screening and carbohydrate antigen 125 (CA 125) for disease and therapy monitoring in ovarian cancer patients. However, their clinical utility is limited by insufficient specificity and sensitivity [53].

## Opportunities of liquid biopsy for cancer predisposition syndromes

### Surveillance and early cancer detection

Representing a defined high risk population, individuals with CPS may have a particularly high benefit from LB-based approaches for effective clinical management and are an important cohort for research and validation of LB strategies. In this regard, one of the most crucial aspects is the early detection of pre-symptomatic cancers, potentially facilitating favorable patient outcome. Currently, surveillance of at-risk individuals is strenuous and often invasive, affecting patients’ quality of life and adherence to protocols. LB-based surveillance would allow non- or minimally-invasive sampling, simultaneously reducing the strain on patients and enabling close cancer surveillance.

Clinical trials and initiatives focusing on early cancer detection in individuals with known cancer predisposition have been initiated. The ‘CHARM: Circulating Tumor DNA in Hereditary And High Risk Malignancies’ (NCT04261972) trial and the ‘EDISYN: Early Detection in Syndromic Neoplasms’ initiative (https://www.edisyn.org) collect and bank cfDNA from CPS patients aiming for ctDNA-based detection of early signs of cancer. Moreover, several clinical trials study the surveillance of CPS patients using already identified LB-analytes. Respective studies have been initiated for hereditary breast cancer, pancreatic and CRC. One trial evaluates the sensitivity and specificity of *TP53* mutations in plasma ctDNA for the diagnosis of new cancer or relapse in *BRCA1* carriers (NCT02608346). The ExoLuminate trial (NCT05625529) focuses on early detection of pancreatic cancer using EV-associated protein biomarkers in patients with an increased risk for pancreatic ductal adenocarcinoma including *BRCA* mutation and LS carriers. Another study (NCT02198092) assesses the suitability of Septin9, a known CRC marker, for CRC detection in individuals with hereditary colon cancer syndromes including LS, Familial adenomatous polyposis (FAP) and MYH-associated polyposis.

In the case of the most common CPS, LS, cancers are characterized by MSI and the detection of this unifying molecular marker by LB may aid to overcome current shortcomings of LS surveillance. Right now, the only recommended surveillance protocol for LS carriers is regular colonoscopy, which was shown to have limited efficacy in preventing cancer. Further, comparable surveillance strategies for LS-associated extracolonic cancers do not exist [8]. Importantly, the cfDNA-based detection of MSI in urine samples from LS cancer patients has recently been reported [54], proving the general suitability of MSI as a LB biomarker. Additionally, LS-associated tumors were found to be characterized by a distinct miRNA expression pattern, which may show potential to be used for LB-based LS surveillance [55], [56]. However, the analysis of these LS-specific markers in peripheral blood is still pending.

### After cancer is before cancer

Cancer biomarkers improve diagnostics and support therapy decisions in clinical oncology. These biomarkers transferred to the LB scenario may offer an alternative dynamic, real-time approach for monitoring disease course. Particularly in patients receiving therapy real-time biomarker detection would enable therapy response monitoring and early detection of potential treatment resistance. This could inform the clinician to adjust therapy in a timely manner to maximize the treatment efficacy and minimize possible side effects.

One of such biomarkers that in the recent years became indispensable in clinical oncology for identifying potential responders to immune checkpoint blockade (ICB) is MSI [57,58]. Presenting with a particularly high mutational load, MSI cancers are more likely to respond to ICB [59]. With the advent of ICB in a neoadjuvant setting [60], MSI analysis from biopsy material guides the selection of likely responders, at the same time carrying major pitfalls, such as tumor heterogeneity, often related to later therapy resistance, and invasive character of the intervention [61]. In contrast, MSI detection in liquid biopsies may offer a possible approach for informing therapy selection without these pitfalls. In fact, it could not only be used as a predictive marker for guiding clinical therapy, but also be utilized for therapy monitoring in MSI cancer patients. Moreover, if proven sufficiently sensitive, this approach could be used to determine minimal residual disease (MRD) in MSI cancer patients. MRD detection is particularly important for MSI patients in the context of LS due to a higher risk for recurrence and secondary malignancies in these patients. Early MRD detection could guide agile treatment strategy adaptations or closer surveillance in this case. However, in the scenario of genetic cancer predisposition, such as LS, it might be required to re-think the value of MRD in the time line of tumor development. Classically, MRD, detects residual cancer material. However, through facilitating mutations genetic cancer predispositions create a favorable milieu for constantly arising and potentially regressing cancer cells. It has been shown that LS carriers present with precancerous lesions that are under tight control of the immune system [15]. Even completely normal colonic mucosa of tumor-free LS carriers presents with pronounced immune infiltration [62]. This strong immune reaction is likely to be one of the major determinants of the limited penetrance of LS.

Thus, individuals with genetic cancer predisposition might present with a higher baseline of MRD compared to individuals at average cancer risk. In this scenario, ‘MRD’ is transferred to ‘before clinical manifestation’ time point, opening the possibility to identify individuals affected by CPS before any clinically detectable disease manifestation. Also here, MSI detection offers a good biomarker for detecting LS-specific baseline ‘MRD’ and any deviations thereof to identify emerging cancer cell clones. However, further research is required to test the performance of LB-based MSI tests for a reliable disease course monitoring.

**Figure 2:**
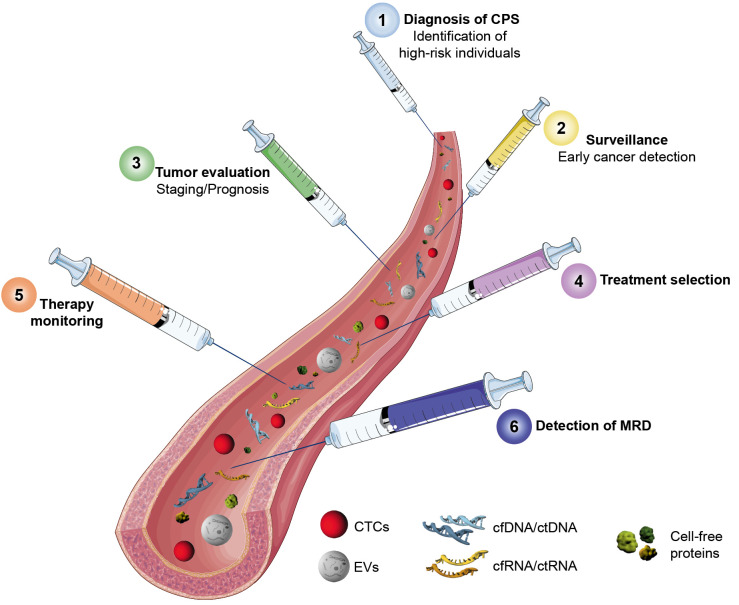
Timeline of potential clinical applications of liquid biopsy in individuals with CPS. CPS: Cancer predisposition syndrome; MRD: Minimal residual disease; CTCs: Circulating tumor cells, EVs: Extracellular vesicles . Parts of the figure were drawn by using pictures from Servier Medical Art. Servier Medical Art by Servier is licensed under a Creative Commons Attribution 3.0 Unported License (https://creativecommons.org/licenses/by/3.0/).

As a mutational process, MMR deficiency results in a certain mutational signature detectable in the somatic genetic alterations of the respective tumors [59]. Mutational signatures, describing the characteristic mutation pattern of the cancer genome, not only provide valuable insight into the evolutionary history of the tumor, but may also guide therapeutic interventions. Besides MMR deficiency-associated signatures that indicate sensitivity to ICB, several signatures were associated with responsiveness to different treatment approaches. For instance, signatures of homologous recombination deficiency are associated with sensitivity to PARP inhibitors [63]. A recent study by Wan et al. demonstrated the feasibility of profiling mutational signatures in plasma using ctDNA [64]. In addition to their therapeutic significance, a minimally invasive screening of mutational signatures might be leveraged for early cancer and MRD detection which would be particularly valuable in individuals with CPS.

## Biological and technical challenges and pitfalls of LB-based approaches

LB-based assays are not fully implemented in routine clinical care due to multiple biological and technical challenges. Generally, the lack of standardized sampling, isolation and characterization protocols for LB analytes can lead to variability and limited applicability of the obtained data. Every LB analyte is further associated with specific challenges.

The main challenge in the use of CTCs is their rarity in peripheral blood, estimates assume one CTC per 10^6^–10^8^ white blood cells [65]. Thus, detection and isolation of CTCs amongst the overwhelming amount of hematological components requires high sensitivity and affinity [65]. Isolation is further complicated by CTCs’ phenotypic and size heterogeneity. For instance, the observed heterogeneous expression of mesenchymal and epithelial markers can affect CTC enrichment technologies dependent on respective surface markers [38]. Besides blood volume, handling and the use of preservatives have to be considered. In addition, time of sampling is relevant, as CTC release is impacted by circadian rhythm [65,66]. By now technical advancements have enabled molecular analyses down to single CTC level, however the required amplification processes render CTC analysis extremely sensitive to contamination. Moreover, the respective analyses are associated with great financial and logistical efforts [38].

Sampling and handling are also critical factors in the analysis of cfDNA/ctDNA. The half-life time of cfDNA/ctDNA is estimated to be 1–2 hours demanding fast isolation and processing or the use of cell membrane-stabilizing preservatives that prevent the lysis of white blood cells and the release of large amounts of cellular DNA [31]. Low ctDNA concentration and its fragmentation pose great technical challenges for downstream analyses. Currently, ctDNA sequencing assays have a limited sensitivity for low abundance mutations with a variant allele frequency below 0.5 % [67]. Notably, inter- and intra-individual variability of ctDNA complicates analyses and demands longitudinal assessments [68].

The main challenge towards the diagnostic use of EVs in LB is heterogeneity of EV subpopulations and the absence of reliable markers distinguishing different EV types. This, plus their nanoscale size, requires technical development enabling efficient and pure isolation of EVs from different bodily fluids [69,70].

## Outlook

While the potential use of LB for surveillance, early detection, therapy monitoring and MRD detection in patients with genetic cancer syndromes is promising, further research and validation are needed to establish its clinical utility. In particular, studies focusing on procedural standardization, evaluation of the clinical benefit, transferability into clinical routine and the analysis and independent validation of sensitivity and specificity, as postulated by the International Liquid Biopsy Standardization Alliance (ILSA) [71], will be necessary. Moreover, cost estimations for implementing liquid biopsy into clinical routine become increasingly relevant and first tools allowing laboratory-specific cost calculations are already available from the COIN Consortium [72]. In summary, with ongoing advancements in technology and increasing understanding of the molecular basis of these syndromes, LB has the potential to revolutionize the clinical management of inherited cancer syndromes, ultimately improving patient outcomes and quality of life.
